# A Colorimetric Probe Based on Functionalized Gold Nanorods for Sensitive and Selective Detection of As(III) Ions

**DOI:** 10.3390/s18072372

**Published:** 2018-07-21

**Authors:** Kun Ge, Jingmin Liu, Guozhen Fang, Peihua Wang, Dongdong Zhang, Shuo Wang

**Affiliations:** 1 State Key Laboratory of Food Nutrition and Safety, College of Food Engineering and Biotechnology, Tianjin University of Science and Technology, Tianjin 300457, China; gekunwlmq@163.com (K.G.); wangpeihua2443@163.com (P.W.); medong@126.com (D.Z.); 2 Tianjin Key Laboratory of Food Science and Health, School of Medicine, Nankai University, Tianjin 300071, China; liujingmin@nankai.edu.cn

**Keywords:** gold nanorods, colorimetric probe, dithiothreitol, arsenic ions

## Abstract

A colorimetric probe for determination of As(III) ions in aqueous solutions on basis of localized surface plasmon resonance (LSPR) was synthesized. The dithiothreitol molecules with two end thiols covalently combined with Au Nanorods (AuNRs) with an aspect ratio of 2.9 by Au-S bond to form dithiothreitol coated Au Nanorods (DTT-AuNRs), acting as colorimetric probe for the determination of As(III) ions. With the adding of As(III) ions, the AuNRs will be aggregated and leading the longitudinal SPR absorption band of DTT-AuNRs decrease due to the As(III) ions can bind with three DTT molecules through an As-S linkage. The potential factors affect the response of DTT-AuNRs to As(III) ions including the concentration of DTT, pH values of DTT-AuNRs, reaction time and NaCl concentration were optimized. Under optimum assay conditions, the DTT-AuNRs colorimetric probe has high sensitivity towards As(III) ions with low detection limit of 38 nM by rules of 3σ/k and excellent linear range of 0.13–10.01 μM. The developed colorimetric probe shows high selectivity for As(III) ions sensing and has applied to determine of As(III) in environmental water samples with quantitative spike-recoveries range from 95.2% to 100.4% with low relative standard deviation of less than 4.4% (*n* = 3).

## 1. Introduction

Arsenic (As) is an inorganic element with high toxicity and occurrence abundant in the environment samples including soil, water, food, rain and vegetations which has attracted great attention worldwide [1,2]. Arsenic has several oxidation states (−3, +3, 0, and +5) in the environment but in water samples it is mostly found as trivalent arsenite or pentavalent arsenate. A number of diseases including cancer, skin damage, problems with cardiovascular, nervous system, respiratory system and blood system are closely related with the continuous intake of As(III) in drinking water [3,4,5]. World Health Organization (WHO) and Environmental Protection Agency (EPA) both set a provisional guideline of 133 nM (10 μg·L^−1^) for maximum arsenic content in ground water in 1993 [6,7]. Several analytical methods have been established to determine the concentration of arsenic in various samples. Many analytical methods with expensive instrumental have been applied to detect arsenic in environmental matrices, such as high performance liquid chromatography combined with inductively coupled plasma mass spectrometry (HPLC–ICP-MS) [8], hydride generation atomic fluorescence spectrometry (HG-AFS) [9], atomic fluorescence spectrometry (AFS) [10], inductively coupled plasma mass spectrometry (ICP-MS) [11], electrochemical method [12,13,14] and atomic absorption spectroscopy (AAS) [15]. Furthermore, various simple, rapid, sensitive and inexpensive methods was also investigated. Fluorescent probe based on CdS quantum dots and Carbon dots have been developed to detect the concentration of arsenic in ground water samples with satisfactory recovery [7,16]. Colorimetric probes based on gold nanoparticles have been also established for determination of arsenic [17,18]. Among these methods, the colorimetric method attract more attention due to the unique merits of simplicity, speediness, sensitivity and low cost.

Recently, gold nanorods has attracted increasing attentions due to the distinctive optical properties of localized surface plasmon resonance (SPR), which possesses two plasmon absorption bands including transverse surface plasmon resonance (transverse SPR) peak and longitudinal surface plasmon resonance (longitudinal SPR) peak produced by plasmon oscillation of short axis and long axis, respectively [19,20,21,22]. The transverse SPR band at about 520 nm and longitudinal SPR band was based on the aspect ratio of AuNRs along with different absorption band from visible to near-infrared region [23,24,25]. On the other hand, various colorimetric probe based on AuNRs due to the surrounding environment will affect the longitudinal SPR peak location and intensity remarkably, and the biological modification of AuNRs surface can also altered the longitudinal SPR peak location and intensity [26,27,28]. Due to the unique optical properties of AuNRs, AuNRs have being applied to various fields, such as biosensing, bioimaging and photodynamic therapy [29,30,31,32,33]. The applications of AuNRs are generally based on functionalization of AuNRs due to the high concentration of cetyltrimethylammonium bromide (CTAB) on the surface of nanorods, which possesses biotoxicity and can maintain the stability of AuNRs [34,35,36]. Considering the above situation, the molecules of modification on surface of AuNRs need strong conjugation ability towards the surface of AuNRs, thiol compounds including cysteine (Cys), dithiothreitol (DTT) and glutathione (GSH) will be ideal candidates due to the fact that the thiol compounds can be covalently combined with AuNRs by Au-S bond instead of cetyltrimethylammonium bromide (CTAB) on the surface of nanorods.

In the past decades, numerous of colorimetric sensors on basis of variation of the longitudinal plasmon absorption of AuNRs for metal ions sensing have been developed. The cysteine (Cys) modified AuNRs (Cys-AuNRs) was developed for Pb^2+^ sensing on basis of the fact that Pb^2+^ ions induce the aggregation of Cys-AuNRs along with decrease of longitudinal surface plasmon resonance absorption peak at 700 nm [37]. The dithiothreitol (DTT) functionalized AuNRs was used as colorimetric probe for determination of Hg^2+^ on the basis of the fact that Cys can induce the aggregation of AuNRs and special affinity of Hg^2+^ to the thiol group for preventing the aggregation of Cys-AuNRs and leading the recovery of longitudinal SPR absorption peak at 650 nm along with an obvious change in color from gray to blue-green [38]. The colorimetric sensors for Cu^2+^ sensing based on Cys-AuNRs was developed. The strong combination of Cu^2+^ with –COOH and –NH_2_ of cysteine leading a stable complex of Cys–Cu–Cys and the Cys-AuNRs will aggregated in the presence of Cu^2+^ along with a rapid and obvious change in color from gray to blue-green [39]. 1-[2-(octylamino) ethyl]-3, 5-diphenylpyrazole (PyL) modified AuNRs (PyL-AuNRs) was developed for determination of Hg^2+^ based on the longitudinal plasmon absorption peak will result in gradual red-shift range from 650 nm to 900 nm and broading the longitudinal plasmon absorption peak range up to 900 nm with obvious change in color from blue-green to gray [40]. A non-aggregation colorimetric sensor based on AuNRs for determination of Cr (VI) was established based on the blue-shift in the presence of Cr (VI) [41]. A glutathione (GSH) modified AuNRs (GSH-AuNRs) has developed for Pb^2+^ sensing based on the aggregation of GSH-AuNRs with the Pb^2+^ adding and result in obvious red-shift [42]. A meso-2, 3-Dimercaptosuccinic acid (DMSA) functionalized gold nanorod on paper has developed for arsenic (III and V) detection in ground water on the basis of aggregation of GNR-PEG-DMSA after arsenic (III and V) addition [43]. The GNR-PEG-DMSA is a colorimetric probe with high sensitivity and excellent selectivity while the preparation of colorimetric probe is also complicated and difficult. Thus, the molecules with a thiol group and have strong affinity to target which can affect the longitudinal SPR of AuNRs is necessary. To our best knowledge, no colorimetric probe for determination of As(III) ions based on functionalized AuNRs with high sensitivity, excellent simplicity and rapidity have been reported so far.

In the present study, we developed a dithiothreitol (DTT) modified AuNRs (DTT-AuNRs) as colorimetric probe for determination of As(III). The UV-Vis absorption spectra of prepared CTAB-AuNRs, AuNRs, and DTT-AuNRs were characterized by UV–vis spectrophotometer. The transmission electron microscopy (TEM) was applied to describe the morphology of AuNRs. The DTT-AuNRs colorimetric probe for sensing As(III) ions shows low limit detection and excellent detection range. The selectivity of proposed colorimetric method was investigated by testing various metal ions including Mn^2+^, Cu^2+^, Cd^2+^, V^3+^, Co^2+^, Ca^2+^, Mg^2+^, Zn^2+^, Cr^3+^, Al^3+^, Hg^2+^, Pb^2+^ and Fe^3+^ by monitoring changes of the longitudinal SPR band. What’s more, the developed colorimetric probe (DTT-AuNRs) was used to determine the concentration of As(III) ions in water samples of environment by quantitative spike recovery method with ideal recoveries. The results of experiment suggested that the proposed colorimetric probe for determination of As(III) ions has some merits including sensitivity, selectivity, simplicity, speediness, and accuracy.

## 2. Materials and Methods

### 2.1. Chemicals

All chemicals used were at least analytically pure. Cetyltrimethylammonium bromide (CTAB, 99%), chloroauric acid (HAuCl_4_·3H_2_O, 99.99%) and ascorbic acid (Vc, 99.99%) were all purchased from Sigma (St. Louis, MO, USA). Dithiothreitol (DTT) and grade heavy metal standard solutions (Mn^2+^, Cu^2+^, Cd^2+^, V^3+^, Co^2+^, Ca^2+^, Mg^2+^, Zn^2+^, Cr^3+^, Al^3+^, Hg^2+^, Pb^2+^ and Fe^3+^) were all purchased from Sinopharm Chemical Reagent Co. Ltd. (Shanghai, China). The buffer was 10 mM HAc-NaAc solution (pH 4.0). Milli-Q purified water was used for all experiments.

### 2.2. Characterization

Transmission electron microscope (TEM, JEM-2010FEF, JEOL, Tokyo, Japan) was applied to characterize the morphology of the AuNRs. The absorption spectra of AuNRs were recorded on Cary50-Bio UV-Vis spectrophotometer (Victoria, Australia) with 1 cm path-length in the wavelength range of 200–1000 nm. The size distribution of AuNRs and DTT-AuNRs exposed to As(III) ions were performed on Mastersizer 2000 (Malvern Instruments Ltd., UK).

### 2.3. Preparation and Modification of AuNRs

Gold nanorods were prepared by seed mediated growth method using the previously published protocol [44]. Briefly, the seed solution was prepared by mixing of 7.5 mL of 0.1 M CTAB with 250 μL of 10 mM gold(III) chloride trihydrate and 600 μL of ice-cold 0.01 M sodium borohydride, then the mixture was stored for 2 h for the next procedure. Subsequently, 4.75 mL of 0.1 M CTAB was added to 200 μL of 10 mM gold(III) chloride trihydrate, then 48 μL of 0.004 M silver nitrate was added into the previous mixture. After a mild mixing of the mixture, 32 μL of ascorbic acid (0.1 M) was added into the previous mixed solution. Finally, 12 μL of the prepared seed solution was mixed with the growth solution in the temperature range of 30–32 °C for at least 24 h for completion of nanorod synthesis. The gold nanorods solution were washed twice by centrifugation at 10,000 rpm/min for 10 min to remove the excess surfactant cetyltrimethylammonium bromide (CTAB) and the final nanorod pellet was dispersed in purified water and stored in the refrigerator at 4 °C.

The AuNRs were modified with dithiothreitol with 1 sulfhydryl group to form DTT-AuNRs. Briefly, the 100 μL of 70 μM dithiothreitol was added dropwise into the 5 mL of 1 nmol·L^−1^ gold nanorods solution (HAc-NaAc buffer, 10 mM, pH = 4.0) followed by mildly stirring for 2 h at room temperature without light and the DTT-AuNRs can maintain the stability at 4 °C in the dark for one week. The concentration of the functionalized AuNRs was calculated to be 1 nM according to the Lambert-Beer law and the extinction coefficient of AuNRs (3.59 × 10^9^ M^−1^·cm^−1^) [45].

### 2.4. Samples

Three kinds of water samples including tap, lake and river water were collected from local water resource. Before the analysis, all environmental water samples were purified by centrifugation at 10,000 rpm/min for 10 min and filtration through a 0.45 μm membrane. The spiked samples were then analyzed immediately without other treatment.

### 2.5. Detection of As(III) Ions by Colorimetric Probe

First, the absorption spectrum of prepared DTT-AuNRs were measured to ensure the photostability of prepared AuNRs in HAc-NaAc buffer solution. Then, 1 mL of different concentrations of As(III) ions were mixed with 500 μL of DTT-AuNRs colorimetric probe with gentle shaking for 10 min and recorded the change of absorption intensity. The selectivity of DTT-AuNRs colorimetric probe to As(III) ions were carried out by adding other metal ions including Mn^2+^, Cu^2+^, Cd^2+^, V^3+^, Co^2+^, Ca^2+^, Mg^2+^, Zn^2+^, Cr^3+^, Al^3+^, Hg^2+^, Pb^2+^ and Fe^3+^ (50 μM) into DTT-AuNRs colorimetric probe with the same experiment conditions.

## 3. Results

### 3.1. Characterization of Functionalized AuNRs

**Figure 1:**
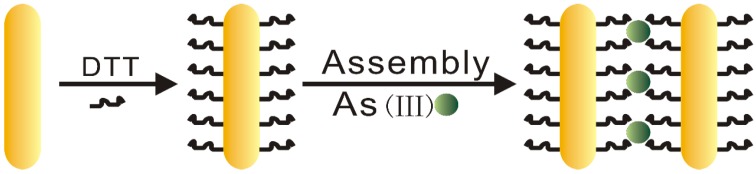
The schematic mechanism of determination of As(III) by DTT-AuNRs colorimetric probe.

Figure 1 shows the mechanism of sensing As(III) ions by proposed DTT-AuNRs colorimetric probe. Firstly, the surface of AuNRs have high concentration of CTAB with positive charge to maintain the stability of AuNRs, thus, a great deal of CTAB surrounding the nanorods surfaces was removed by centrifugation and leave behind huge area with low concentration CTAB on the long sides of AuNRs surfaces, then one end thiol of the DTT covalently combined with AuNRs by Au-S bond instead of the CTAB layer surrounding the nanorods surfaces. The proposed colorimetric method for selective detection of As(III) ions is on the basis that the As(III) ions can bind with three DTT-AuNRs through an As-S linkage and induce the aggregation of DTT-AuNRs. 

**Figure 2:**
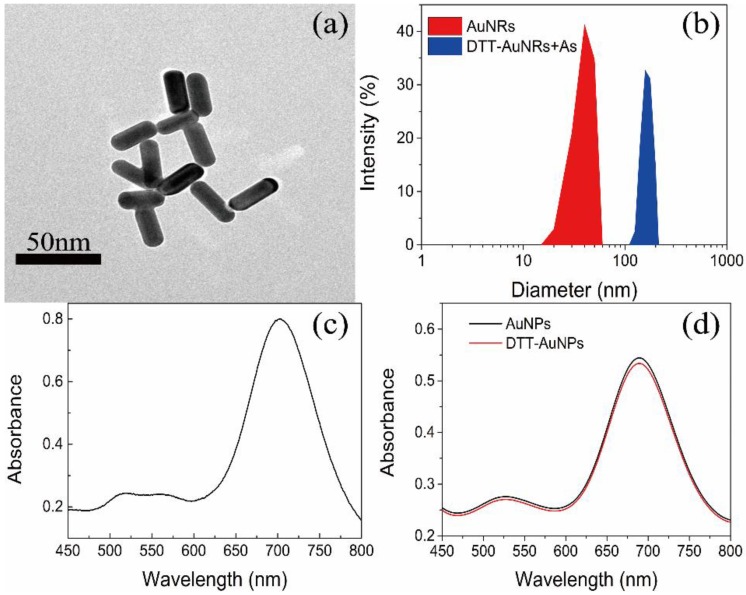
(**a**) TEM image of AuNRs; (**b**) Size distribution of AuNRs and DTT-AuNRs exposed to As(III); (**c**) UV–vis absorption spectra of CTAB-coating AuNRs; (**d**) UV–vis absorption spectra of AuNRs and DTT-AuNRs.

**Figure 3:**
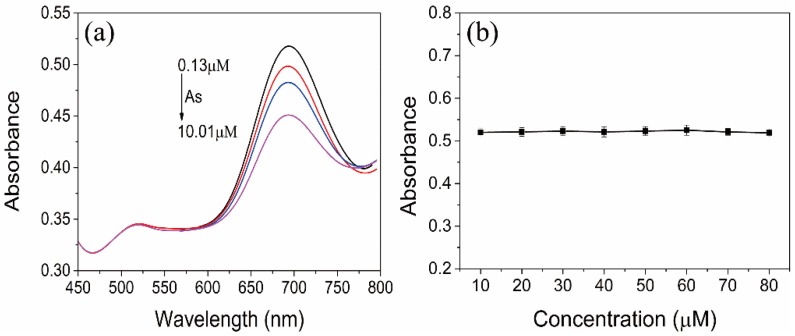
(**a**) UV–vis absorption spectra of DTT-AuNRs (0.8 nM) upon adding increasing concentration of As(III) ions (from 0.13 to 10.01 μM); (**b**) Effects of concentration of DTT on UV-vis spectra of AuNRs.

The transmission electron microscopy (TEM) images are applied to characterize the morphology and UV–vis absorption spectroscopy is applied to confirm the spectral properties. The transmission electron microscopy (TEM) images of AuNRs were showed in Figure 2a, we can note that the separated AuNR shows well-shared shape of rod with the aspect ratio of 2.9 ± 0.1. The AuNRs have distinctive optical characteristics that they have 2 typical absorption peaks, the transverse SPR peak at about 520 nm and the longitudinal SPR peak at about 700 nm (Figure 2c). As showed in Figure 2d, after removing the CTAB surrounding the nanorods surfaces and modification of DTT, the longitudinal SPR peak showed slight blue-shift compared to AuNRs and DTT-AuNRs, and the longitudinal SPR peak of DTT-AuNRs have lower absorption peak than AuNRs, indicating that that dithiothreitol molecules are mainly conjugated on the long sides of AuNRs surface. On the other hand, the band change only occur in longitudinal SPR instead of transverse SPR indicating that DTT molecules are majorly combined on the long sides of AuNRs surfaces based on strong affinity between Au with -SH. Furthermore, size distribution of AuNRs and DTT-AuNRs exposed to As(III) further shows that the DTT-AuNRs were aggregated after adding the As(III) ions into DTT-AuNRs colorimetric probe (Figure 2b). Moreover, under optimal conditions, the longitudinal SPR peak will decrease gradually with the increase of concentration of As(III) ions (Figure 3a).

### 3.2. Stability Evaluation of AuNRs

**Figure 4:**
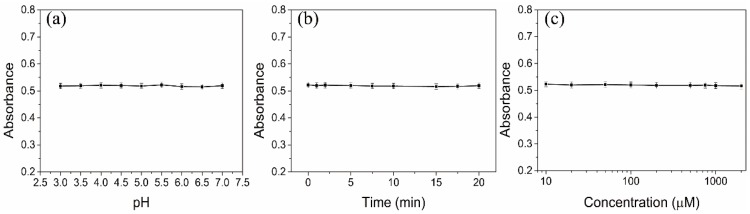
Evaluation of stability of DTT-AuNRs colorimetric probe: (**a**) Effect of the pH value of NaAc-HAc buffer (10 mM); (**b**) Effect of the reaction time; (**c**) Effect of the concentration of NaCl.

In addition, in order to evaluate the stability of the DTT-AuNRs colorimetric probe to ensure the sensitive and selective sensing performance, some experiment conditions including pH values, reaction time and concentration of NaCl were carried out by recording the absorption value. As showed in Figure 4a, NaAc-HAc buffer (10 mM) with pH values range of 3.0–7.0 can keep the stability of DTT-AuNRs. Moreover, the reaction time produced negligible effect to the absorption value of DTT-AuNRs within 20 min. In addition, the concentration of NaCl range of 10–2000 μM also show high stability of DTT-AuNRs. The above results means that the as-prepared the DTT-AuNRs colorimetric probe have excellent stability in the absence of As(III) ions which can prove that the colorimetric probe will maintain high stability and uniformity before As(III) ions addition.

**Figure 5:**
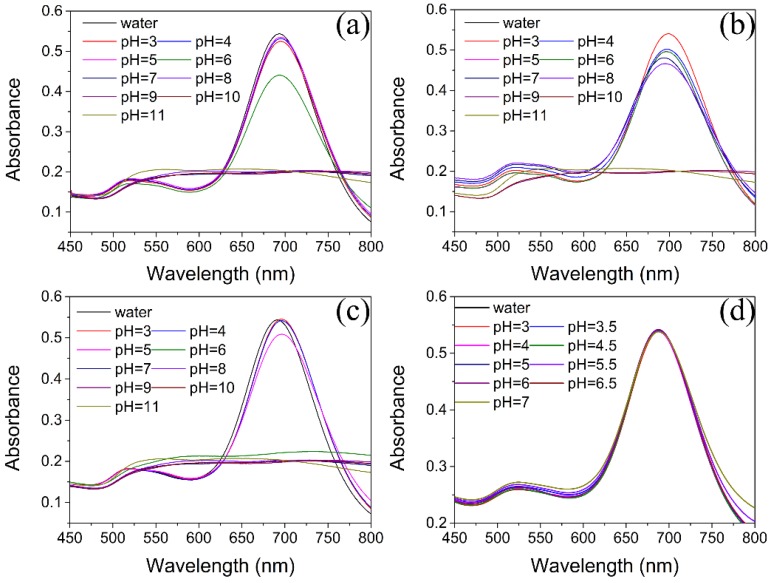
Effects of pH and buffer media on UV-vis spectra of AuNRs: (**a**) 10 mM PBS; (**b**) 10 mM Tris-HCl; (**c**) BR buffer; (**d**) 10 mM NaAc-HAc.

In order to choose an appropriate buffer solution for ensuring the good stability of AuNRs, the UV–vis spectra of AuNRs based on different pH values in various buffer media was evaluated. In phosphate buffer solution (PBS), the longitudinal SPR absorbance of AuNRs decreased significantly at pH > 6.0 (Figure 5a). The same phenomenon can be found in Tris-HCl and *Br*itton-Robinson buffer at pH > 6.0 and pH > 5.0, respectively (Figure 5b,c). Moreover, the AuNRs also shows different stability at low pH among PBS, Tris-HCl and *Br*itton-Robinson buffer and no buffer can maintain the same longitudinal SPR absorbance at low pH condition. The decrease of the longitudinal SPR absorbance of AuNRs at high pH suggests the serious aggregation of AuNRs and indicates that the AuNRs can maintain the stability under acidic pH condition due to the existence of CTAB on the surface of AuNRs. The CTAB is a kind of quaternary ammonium salts with positive charge under acidic conditions which can provide excellent effect of electrostatic protection to maintain the stability of AuNRs and prevent aggregation. However, under high pH conditions, the protective effect of CTAB to AuNRs will be weaken, resulting in instability of AuNRs and decrease of longitudinal SPR absorption peak. Therefore, the appropriate buffer medium to confirm the stability of the AuNRs solution under low concentration conditions of CATB is necessary absolutely. We find that the NaAc-HAc buffer (10 mM) with pH values range of 3.0–7.0 can keep the stability of AuNRs solution with low concentration of CTAB (Figure 5d), thus, the NaAc-HAc buffer (10 mM) was chosen the final media for further experiments. Moreover, the concentration of DTT range of 10–80μM produces negligible effect to the absorption value of AuNRs (Figure 3b), which can ensure that the high stability of AuNRs in different concentration of DTT.

### 3.3. Optimization of Experimental Conditions

To further achieve sensitive and selective sensing performance, some optimal experiment conditions including concentration of DTT, pH values, reaction time and concentration of NaCl were optimized.

#### 3.3.1. Effect of DTT Concentration

**Figure 6:**
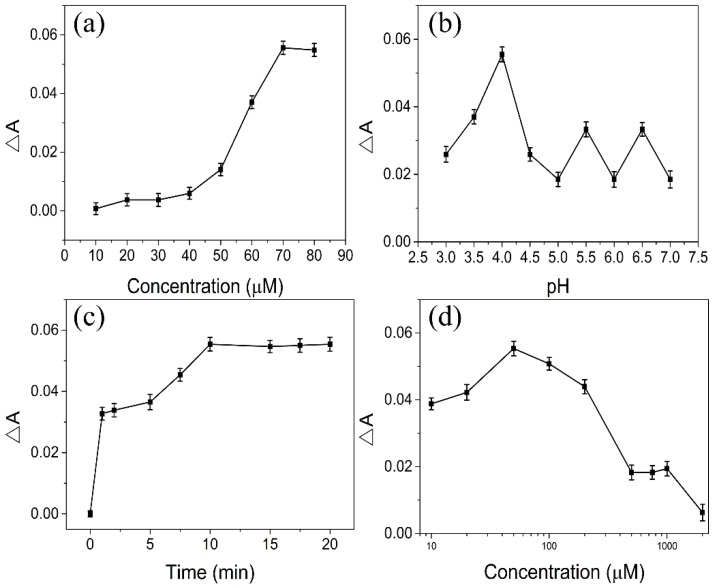
Optimization of developed AuNR probe for the colorimetric detection of As(III): (**a**) Effect of the concentration of DTT; (**b**) Effect of the pH value of NaAc-HAc buffer (10 mM); (**c**) Effect of the reaction time; (**d**) Effect of the concentration of NaCl.

The concentration of DTT will affect the sensitivity of sensing As(III) ions, a low concentration of DTT will show weak response to As(III) ions and high concentration of DTT will induce the aggregation of AuNRs, thus the different concentration of DTT was optimized. As showed in Figure 6a, the ΔA of DTT–AuNRs system increases slowly with the increase of DTT concentration within 50 μM and increases significantly upon the concentration of DTT reaches to 70 μM in the presence of 6.67 μM As(III) ions. Therefore, the final concentration of DTT was chosen 70 μM to modify AuNRs.

#### 3.3.2. Effect of pH

The pH values of DTT-AuNRs colorimetric probe plays an important role in sensing As(III). Therefore, the pH of DTT-AuNRs sensing system was investigated by 10 mM NaAc-HAc buffer range of 3.0–7.0 due to the NaAc-HAc buffer can maintain the stability of DTT-AuNRs sensing system. As displayed in Figure 6b, the ΔA of DTT–AuNRs system increases slowly with the increase of pH values within 4.0 and ΔA of DTT–AuNRs system decreases significantly while the pH values exceed 4.0 in the presence of 6.67 μM As(III) ions. As mentioned above, high pH values can induce the aggregation of AuNRs and too low pH values may affect the determination of As(III) ions. So, 10 mM NaAc-HAc buffer of pH = 4.0 was chosen as the final reaction media.

#### 3.3.3. Effect of Reaction Time

The reaction time between As(III) ions and DTT-AuNRs colorimetric probe also affects the sensitivity of As(III) ions, if the reaction time is too short will result in incomplete reaction between As(III) ions and DTT-AuNRs and too long time seems to be a waste of time. Thus, the effect of incubation time between DTT-AuNRs colorimetric probe and As(III) ions was tested by recording the change of longitudinal SPR band absorption (ΔA) in the presence of 6.67 μM As(III) ions. As showed in Figure 6c, the ΔA values increase significantly at the beginning and increase slowly range from 1–10 min, while the ΔA values increases with the increase of incubation time and slightly changes in the presence of 6.67 μM As(III) ions when reaction time is longer than 10 min. In order to achieve maximum ΔA values between As(III) ions and DTT-AuNRs colorimetric probe, 10 min was chosen the final reaction time.

#### 3.3.4. Effect of NaCl Concentration

The concentration of NaCl is also plays an important role in in sensing As(III). NaCl can affects the stability of DTT-AuNRs colorimetric probe due to the fact that high concentration of NaCl can induce the aggregation of AuNRs. Thus, the effect of concentration of NaCl on the proposed colorimetric probe was evaluated with range of 10 μM–2 mM (Figure 6d). The ΔA values increases with the increase of NaCl concentration and obviously changes in the presence of 6.67 μM As(III) ions when NaCl concentration is lower than 50 μM. The ΔA values of DTT-AuNRs colorimetric probe reaches maximum at 50 μM NaCl and the ΔA values decreases with the increase of NaCl concentration while the NaCl concentration is higher than 50 μM. Thus, 50 μM NaCl was chosen the final added concentration of salt.

### 3.4. Selectivity

**Figure 7:**
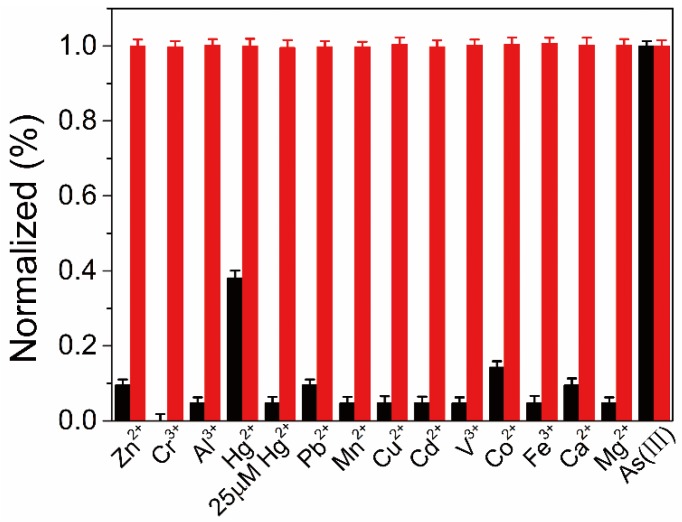
Selectivity test of the developed colorimetric probe for As(III) ions (6.67 μM) over other metal ions (50 μM, except for Hg^2+^, 25 μM). Black bars denote the responses of individual metal ions, while red bars show the responses of As(III) (6.67 μM) in the presence of other metal ions.

The selectivity of the developed colorimetric probe was evaluated under the same experiment conditions for As(III) determination and 13 kinds of interfering metal ions were used to selectivity test. Figure 7 showed that 13 kinds of chosen metal ions exerted negligible effect to DTT-AuNRs colorimetric probe compared to 6.67 μM As(III) at 50 μM level, suggesting the high specificity of the proposed DTT-AuNRs colorimetric probe for determination of As(III) ions. Furthermore, the interference assay was investigated by mixing the As(III) ions (6.67 μM) with 50 μM other 13 kinds of metal ions and recording the changes of longitudinal SPR absorption peak. No obvious changes in UV-vis absorption spectrum of DTT-AuNRs colorimetric probe to As(III) ions in the absence and in the presence of above interferential metal ions except Hg^2+^ (25 μM) was noted. The response of DTT-AuNRs colorimetric probe to Hg^2+^ may due to the special affinity of Hg^2+^ to the thiols and Hg^2+^ ions will detaching DTT from DTT-AuNRs and the interference of Hg^2+^ can be reduced or eliminated by decreasing the concentration of Hg^2+^ from 50 μM to 25 μM which is still higher than the maximum detectable concentration of As(III) ions in developed colorimetric probe mentioned above.

### 3.5. Determination of As(III)

**Figure 8:**
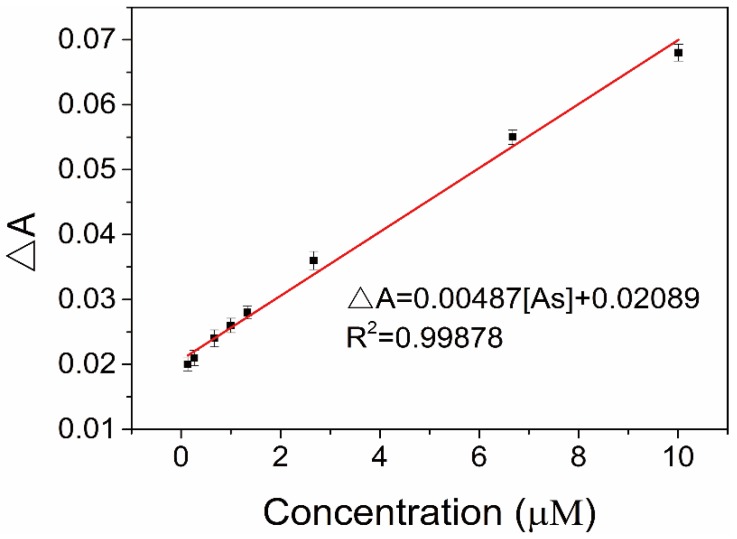
Plot of decreased longitudinal SPR absorption intensity (ΔA) against As(III) concentration over the linear range of 0.13–10.01 μM.

Under the optimal conditions, the linearity and detection limit of DTT-AuNRs colorimetric probe were constructed. As displayed in Figure 8, after adding increasing concentration of As(III) ions into DTT-AuNRs colorimetric probe in NaAc-HAc buffer at pH 4.0 result in an obvious decrease of the longitudinal SPR absorption intensity of DTT-AuNRs colorimetric probe due to the strong and specific affinity of As(III) ions to the dithiothreitol and a linear relationship between the ΔA of the DTT-AuNRs colorimetric probe, and the linear equation ΔA = 0.00487 [As] + 0.02089 with a correlation coefficient R^2^ = 0.99878 was observed, the calibration plot exhibits an excellent detection range of 0.13–10.01 μM, in addition, the limit of detection (LOD) was estimated to be 38 nM by rules of 3 σ/k. The above results suggest that the proposed DTT-AuNRs colorimetric probe have huge potential for determination of As(III) ions in actual sample with low concentration level.

### 3.6. Analysis of Samples

The analytical figures of highlight for proposed DTT-AuNRs colorimetric probe for As(III) sensing under optimal assay conditions are summarized in Table 1. The developed colorimetric probe have an excellent detection range from 0.13 to 10.01 μM, and the limit of detection (LOD) can reaches 38 nM (3 σ/k) which is lower the maximum permissible maximum concentration levels of 0.133 μM (10 ppb) developed by World Health Organization (WHO) and Environmental Protection Agency (EPA). On the other hand, to evaluate the precision of DTT-AuNRs colorimetric probe for the determination of As(III) ions, the relative standard deviation (RSD) was investigated by operating 11 repeated measurements of 2 μM As(III) ions and can reaches 2.1%, which indicating high reliability of this developed colorimetric method.

The developed DTT-AuNRs colorimetric probe was also applied to detect the As(III) in environmental water samples by quantitative spike recovery method. As showed in Table 2, 3 kinds of water samples collected locally were used to determination for As(III) ions. The developed DTT-AuNRs colorimetric probe produced negligible effect to the longitudinal SPR absorption peak when the spiked concentration of As(III) is 0 and the quantitative spike recoveries for the detection of As(III) ions by DTT-AuNRs colorimetric probe ranged from 95.2% to 100.4% with low relative standard deviation of less than 4.4% (*n* = 3). To our best knowledge, the proposed DTT-AuNRs colorimetric probe have excellent potentials for determination of As(III) in real samples.

## 4. Conclusions

In conclusion, we have successfully developed a simple, rapid, selective and sensitive colorimetric method for determination of As(III) ions based on AuNRs with an aspect ratio of 2.9. The detection strategy that As(III) ions can induce the aggregation of AuNRs and leading the decrease of longitudinal SPR absorption peak due to the strong coordination ability of DTT with As(III) ions. Notably, the proposed method shows high sensitivity, high selectivity and excellent performance in the detection of environmental water samples by spectrophotometry and without intricate process and special instruments. Under the optimal assay conditions, the DTT-AuNRs colorimetric probe for determination of As(III) ions has lowly limit detection (LOD = 38 nM) and highly selectivity toward As(III) ions, with an excellent liner detection range from 0.13 to 10.01 μM. What’s more, the proposed DTT-AuNRs colorimetric probe was applied to determine the amount of As(III) ions in environmental water samples with good recoveries of 95.2% to 100.4% with low RSD of less than 4.4%. This work has proposed a new perspective for detection of As(III) ions in real samples and has the good potential for monitoring the pollution conditions of metal ions.

## Figures and Tables

**Table 1:** Analytical figures of merit of the proposed AuNRs colorimetric probe for As(III).

Detection limit (3*s*)/nM	38
Linear range/μM	0.13–10.01
Calibration function (As, conc./μM)	ΔA = 0.00487 [As] + 0.02089
Correlation coefficient (γ^2^)	0.99878
Precision (RSD, *n* = 11) (%)	2.1 (2 μM)

**Table 2:** Analytical results for the determination of As(III) in environmental water samples by proposed colorimetric probe.

Sample	Added Amount (μM)	Concentration (Mean ± s, *n* = 3/μM)	Recovery (Mean ± s, *n* = 3) (%)
Tap water	0	Not detectable	/
	3	2.86 ± 0.12	95.2 ± 4.0
	5	4.95 ± 0.17	99.0 ± 3.4
Lake water	0	Not detectable	/
	3	2.95 ± 0.04	98.4 ± 1.4
	5	5.02 ± 0.10	100.4 ± 1.9
River water	0	Not detectable	/
	3	2.93 ± 0.13	95.4 ± 4.3
	5	4.88 ± 0.19	97.7 ± 3.9

## References

[1] Sun H.J., Rathinasabapathi B., Wu B., Luo J., Pu L.P., Ma L.Q. (2014). Arsenic and selenium toxicity and their interactive effects in humans. Environ. Int..

[2] Vermehren J., Polta A., Zimmermann O., Herrmann E., Poynard T., Hofmann W.P., Bojunga J., Sarrazin C., Zeuzem S., Friedrich-Rust M. (2012). Comparison of acoustic radiation force impulse imaging with transient elastography for the detection of complications in patients with cirrhosis. Liver Int..

[3] Ma J., Sengupta M.K., Yuan D., Dasgupta P.K. (2014). Speciation and detection of arsenic in aqueous samples: A review of recent progress in non-atomic spectrometric methods. Anal. Chim. Acta.

[4] Rager J.E., Bailey K.A., Smeester L., Miller S.K., Parker J.S., Laine J.E., Drobná Z., Currier J., Douillet C., Olshan A.F. (2014). Prenatal arsenic exposure and the epigenome: Altered microRNAs associated with innate and adaptive immune signaling in newborn cord blood. Environ. Mol. Mutagen..

[5] Rizwan S., Naqshbandi A., Farooqui Z., Khan A.A., Khan F. (2014). Protective effect of dietary flaxseed oil on arsenic-induced nephrotoxicity and oxidative damage in rat kidney. Food Chem. Toxicol..

[6] Mulvihill M., Tao A., Benjauthrit K., Arnold J., Yang P. (2008). Surface-enhanced Raman spectroscopy for trace arsenic detection in contaminated water. Angew. Chem. Int. Ed. Engl..

[7] Gupta A., Verma N.C., Khan S., Nandi C.K. (2016). Carbon dots for naked eye colorimetric ultrasensitive arsenic and glutathione detection. Biosens. Bioelectron..

[8] Liu X., Zhang W., Hu Y., Cheng H. (2013). Extraction and detection of organoarsenic feed additives and common arsenic species in environmental matrices by HPLC–ICP-MS. Microchem. J..

[9] Chen B., Corns W.T., Stockwell P.B., Huang J.-H. (2014). Accurate fast screening for total and inorganic arsenic in rice grains using hydride generation atomic fluorescence spectrometry (HG-AFS). Anal. Methods.

[10] Fang Y., Sun X., Yang W., Ma N., Xin Z., Fu J., Liu X., Liu M., Mariga A.M., Zhu X. (2014). Concentrations and health risks of lead, cadmium, arsenic, and mercury in rice and edible mushrooms in China. Food Chem..

[11] Jackson B., Liba A., Nelson J. (2015). Advantages of reaction cell ICP-MS on doubly charged interferences for arsenic and selenium analysis in foods. J. Anal. At. Spectrom..

[12] Jena B.K., Raj C.R. (2008). Gold nanoelectrode ensembles for the simultaneous electrochemical detection of ultratrace arsenic, mercury, and copper. Anal. Chem..

[13] Kim U., Vandergiessen J., Savarimuthu X. Implementation of electrochemical sensors in arsenic-contaminated areas of West Bengal in India toward rapid and point-of-use detection of arsenic in drinking water. Proceedings of the Global Humanitarian Technology Conference.

[14] Ramesha G.K., Sampath S. (2011). In-situ formation of graphene–lead oxide composite and its use in trace arsenic detection. Sens. Actuators B Chem..

[15] Linhart O., Smolejová J., Červený V., Hraníček J., Nováková E., Resslerová T., Rychlovský P. (2016). Determination of As by UV-photochemical generation of its volatile species with AAS detection. Monatsh. Chem. Chem. Mon..

[16] Butwong N., Noipa T., Burakham R., Srijaranai S., Ngeontae W. (2011). Determination of arsenic based on quenching of CdS quantum dots fluorescence using the gas-diffusion flow injection method. Talanta.

[17] Shrivas K., Shankar R., Dewangan K. (2015). Gold nanoparticles as a localized surface plasmon resonance based chemical sensor for on-site colorimetric detection of arsenic in water samples. Sens. Actuators B Chem..

[18] Kalluri J.R., Arbneshi T., Afrin Khan S., Neely A., Candice P., Varisli B., Washington M., McAfee S., Robinson B., Banerjee S. (2009). Use of Gold Nanoparticles in a Simple Colorimetric and Ultrasensitive Dynamic Light Scattering Assay: Selective Detection of Arsenic in Groundwater. Angew. Chem..

[19] Zhang J., Sun Y., Xu B., Zhang H., Gao Y., Zhang H., Song D. (2013). A novel surface plasmon resonance biosensor based on graphene oxide decorated with gold nanorod-antibody conjugates for determination of transferrin. Biosens. Bioelectron..

[20] Wang X., Li Y., Wang H., Fu Q., Peng J., Wang Y., Du J., Zhou Y., Zhan L. (2010). Gold nanorod-based localized surface plasmon resonance biosensor for sensitive detection of hepatitis B virus in buffer, blood serum and plasma. Biosens. Bioelectron..

[21] Tong L., Wei Q., Wei A., Cheng J.X. (2009). Gold nanorods as contrast agents for biological imaging: Optical properties, surface conjugation and photothermal effects. Photochem. Photobiol..

[22] Alkilany A.M., Thompson L.B., Boulos S.P., Sisco P.N., Murphy C.J. (2012). Gold nanorods: Their potential for photothermal therapeutics and drug delivery, tempered by the complexity of their biological interactions. Adv. Drug Deliv. Rev..

[23] Wang C., Chen Y., Wang T., Ma Z., Su Z. (2007). Biorecognition-Driven Self-Assembly of Gold Nanorods: A Rapid and Sensitive Approach toward Antibody Sensing. Chem. Mater..

[24] Huang X., El-Sayed I.H., Qian W., El-Sayed M.A. (2007). Cancer cells assemble and align gold nanorods conjugated to antibodies to produce highly enhanced, sharp, and polarized surface Raman spectra: A potential cancer diagnostic marker. Nano Lett..

[25] Huang X., Jain P.K., Elsayed I.H., Elsayed M.A. (2007). Gold nanoparticles: Interesting optical properties and recent applications in cancer diagnostics and therapy. Nanomedicine.

[26] Huang H., Qu C., Liu X., Huang S., Xu Z., Zhu Y., Chu P.K. (2011). Amplification of localized surface plasmon resonance signals by a gold nanorod assembly and ultra-sensitive detection of mercury. Chem. Commun..

[27] Liang G.X., Wang L., Zhang H., Han Z., Wu X. (2012). A colorimetric probe for the rapid and selective determination of mercury(II) based on the disassembly of gold nanorods. Microchim. Acta.

[28] Rex M., Hernandez F.E., Campiglia A.D. (2006). Pushing the limits of mercury sensors with gold nanorods. Anal. Chem..

[29] Pissuwan D., Valenzuela S.M., Cortie M.B. (2008). Prospects for Gold Nanorod Particles in Diagnostic and Therapeutic Applications. Biotechnol. Genet. Eng. Rev..

[30] And C.Y., Irudayaraj J. (2007). Multiplex Biosensor Using Gold Nanorods. Anal. Chem..

[31] Mayer K.M., Lee S., Liao H., Rostro B.C., Fuentes A., Scully P.T., Nehl C.L., Hafner J.H. (2008). A Label-Free Immunoassay Based Upon Localized Surface Plasmon Resonance of Gold Nanorods. ACS Nano.

[32] Li P.C., Shieh D.B., Wang C.R., Wei C.W., Liao C.K., Ding A.A., Wu Y.N., Poe C., Jhan S. (2008). In vivo photoacoustic molecular imaging with simultaneous multiple selective targeting using antibody-conjugated gold nanorods. Opt. Express.

[33] Singh A.K., Senapati D., Wang S., Griffin J., Neely A., Candice P., Naylor K.M., Varisli B., Kalluri J.R., Ray P.C. (2009). Gold Nanorod Based Selective Identification of Escherichia coli Bacteria Using Two-Photon Rayleigh Scattering Spectroscopy. ACS Nano.

[34] Liu X., Dai Q., Austin L., Coutts J., Knowles G., Zou J., Chen H., Huo Q. (2008). A one-step homogeneous immunoassay for cancer biomarker detection using gold nanoparticle probes coupled with dynamic light scattering. J. Am. Chem. Soc..

[35] Zhang Z., Wang L., Wang J., Jiang X., Li X., Hu Z., Ji Y., Wu X., Chen C. (2012). Mesoporous silica-coated gold nanorods as a light-mediated multifunctional theranostic platform for cancer treatment. Adv. Mater..

[36] Von Maltzahn G., Park J.H., Agrawal A., Bandaru N.K., Das S.K., Sailor M.J., Bhatia S.N. (2009). Computationally guided photothermal tumor therapy using long-circulating gold nanorod antennas. Cancer Res..

[37] Cai H.-H., Lin D., Wang J., Yang P.-H., Cai J. (2014). Controlled side-by-side assembly of gold nanorods: A strategy for lead detection. Sens. Actuators B Chem..

[38] Bi N., Chen Y., Qi H., Zheng X., Chen Y., Liao X., Zhang H., Tian Y. (2012). Spectrophotometric determination of mercury(II) ion using gold nanorod as probe. Sens. Actuators B Chem..

[39] Liu J.M., Wang H.F., Yan X.P. (2011). A gold nanorod based colorimetric probe for the rapid and selective detection of Cu^2+^ ions. Analyst.

[40] Placido T., Aragay G., Pons J., Comparelli R., Curri M.L., Merkoci A. (2013). Ion-directed assembly of gold nanorods: A strategy for mercury detection. ACS Appl. Mater. Interfaces.

[41] Li F.-M., Liu J.-M., Wang X.-X., Lin L.-P., Cai W.-L., Lin X., Zeng Y.-N., Li Z.-M., Lin S.-Q. (2011). Non-aggregation based label free colorimetric sensor for the detection of Cr (VI) based on selective etching of gold nanorods. Sens. Actuators B Chem..

[42] Durgadas C.V., Lakshmi V.N., Sharma C.P., Sreenivasan K. (2011). Sensing of lead ions using glutathione mediated end to end assembled gold nanorod chains. Sens. Actuators B Chem..

[43] Priyadarshni N., Nath P., Nagahanumaiah, Chanda N. (2018). DMSA-Functionalized Gold Nanorod on Paper for Colorimetric Detection and Estimation of Arsenic (III and V) Contamination in Groundwater. ACS Sustain. Chem. Eng..

[44] Joshi P.P., Yoon S.J., Hardin W.G., Emelianov S., Sokolov K.V. (2013). Conjugation of Antibodies to Gold Nanorods through Fc Portion: Synthesis and Molecular Specific Imaging. Bioconjug. Chem..

[45] Orendorff C.J., Murphy C.J. (2006). Quantitation of metal content in the silver-assisted growth of gold nanorods. J. Phys. Chem. B.

